# The Phylogenetic Implications of the Mitochondrial Genomes of *Macropsis notata* and *Oncopsis nigrofasciata*

**DOI:** 10.3389/fgene.2020.00443

**Published:** 2020-05-20

**Authors:** Jia-Jia Wang, Yun-Fei Wu, Mao-Fa Yang, Ren-Huai Dai

**Affiliations:** The Provincial Key Laboratory for Agricultural Pest Management Mountainous Region, Institute of Entomology, Guizhou University, Guiyang, China

**Keywords:** leafhopper, *Macropsis notata*, *Oncopsis nigrofasciata*, mitogenome, phylogenetic analyses

## Abstract

Macropsinae are forest pests that feed on woody plants. They can damage the growth of trees and crops, and some species can also spread plant pathogens. Due to their widespread effects, these leafhoppers are of great economic significance, which is why there is a need to study their genomes. To fill the gap in the mitochondrial genomic data of the subfamily Macropsinae, we sequenced the complete mitochondrial genomes of *Macropsis notata* and *Oncopsis nigrofasciata* (which were 16,323 and 15,927 bp long, respectively). These two species are representative species of the leafhoppers group (Cicadellidae); the mitochondrial genomes of these species range from a length of 15,131 bp (*Trocnadella arisana*) to 16,811 bp (*Parocerus laurifoliae*). Both mitogenomes contained 37 typical insect mitochondrial genes and a control region; there were no long non-coding sequences. The genes within the mitogenome were very compact. The mitogenomes from both species contained two kinds of parallel repeat units in the control region. The whole mitogenomes of Macropsinae showed a heavy AT nucleotide bias (*M. notata* 76.8% and *O. nigrofasciata* 79.0%), a positive AT Skew (0.15 and 0.12), and a negative GC Skew (–0.14 and –0.08). Upon comparative ML and BI analysis, some clade relationships were consistent among the six trees. Most subfamilies were reconstructed into monophyletic groups with strong support in all analyses, with the exception of Evacanthinae and Cicadellinae. Unlike the results of previous research, it was shown that although all Deltocephalinae species are grouped into one clade, they were not the sister group to all other leafhoppers. Further, Cicadellinae and Evacanthinae were occasionally reconstructed as a polyphyletic and a paraphyletic group, respectively, possibly due to the limited numbers of samples and sequences. This mitogenome information for *M. notata* and *O. nigrofasciata* could facilitate future studies on the mitogenomic diversity and evolution of the related Membracoidea, and eventually help to control their effects on plants for the betterment of society at large.

## Introduction

Macropsinae of the family Cicadellidae, Auchenorrhyncha, and order Hemiptera, are distributed worldwide. Currently, more than 750 species of 19 genera in this subfamily have been reported globally; in China, 108 species of 7 genera are found. Macropsinae leafhoppers mostly feed on woody plants and are ecologically and economically important forest pests. Families that are currently known to host them include: Berberidaceae, Betulaceae, Elaeagnaceae, Fagaceae, Rosaceae, Salicaceae, and Ulmaceae (Li et al., 2012, 2013, 2014). The previous studies that were conducted on Macropsinae were mainly focused on the discovery of new species and the discussion on their taxonomic status. However, there are no studies on the molecular phylogenetics of Macropsinae, except for a preliminary study on the relationships between 25 species of Macropsinae based on *COI* fragments (Li and Dai, 2018). Since they can feed on plant juices, damage the growth of trees and crops, cause direct harm, and even spread plant pathogens, these leafhoppers are of great economic significance. According to previous studies, five species of Macropsinae are capable of transmitting plant pathogens (Harris and Maramorosch, 1979; Carraro et al., 2004). In previous phylogenetic studies within Cicadellidae, the relationships between each subfamily and the morphological characteristics of the members of this family have not been studied in detail. Therefore, there is an economic need for more studies centered around the *Macropsinae* species.

With advancements in bioinformatics and sequencing technologies, the mitogenome is being widely used in the molecular, evolutionary, phylogenetic, and population genetic studies of insects (Li et al., 2017; Song et al., 2017; Liu et al., 2018, 2019; Song et al., 2019). The mitogenome of leafhoppers is a typical circular, double-stranded DNA molecule, about 14.5–17 kb in length; it contains 37 typical mitochondrial genes (13 protein coding, 22 transfer RNA, and 2 ribosomal RNA genes), and a long non-coding region (control region) (Cameron, 2014; Wang et al., 2015, 2018; Wu et al., 2016; Zhou et al., 2016; Yu et al., 2017). Until now, 106 complete or partial mitogenome sequences can be found for leafhoppers in GenBank (Supplementary Table S2), and nearly half of them are only identified on a genus or even subfamily level. We randomly selected two common species of Macropsinae [*Macropsis notata* (the host is willow) and *Oncopsis nigrofasciata* (the host is birch)] to sequence and annotate their mitogenomes, in order to better understand their mitogenomic characteristics and phylogenetic relationships within this group. We hope that two mitogenome sequences of Macropsinae in this study will be valuable for research on the identification and phylogenetic analysis of leafhoppers.

## Materials and Methods

### Sample Collection and DNA Extraction

The sample collection information is provided in Supplementary Table S1. Live specimens were preserved in 100% ethanol and stored at –20°C until identification and DNA extraction. Samples were identified by their morphological characteristics (Li et al., 2012, 2019). Genomic DNA was extracted from adult specimens using the Qiagen DNeasy© Tissue kit according to the manufacturer’s protocol. Voucher DNA and other specimens were deposited at the Institute of Entomology, Guizhou University, Guiyang, China.

Reference sequences of COI fragments (600 bp) were amplified by universal primers of insects (primers from Folmer et al., 1994, LCO1490, GGTCAACAAATCATAAAGATATTGG, and HCO2198, TAAACTTCAGGGTGACCAAAAAATCA). PCR amplification was conducted using the PCR MasterMix (Tiangen Biotech Co. Ltd., Beijing, China) according to the manufacturer’s manual. The amplification conditions were as follows: pre-denaturation step for 3 min at 94°C; 30 cycles of denaturation at 94°C for 30 s, 50°C for 30 s, and elongation at 70°C for 1 min; and an additional elongation step at 70°C for 8 min, and direct sequencing of PCR products by Sangon Biotech (Shanghai) Co., Ltd. Sequences were searched through BLAST.^1^ The sequences had a similarity of at least 100%, as verified by NCBI (*Macropsis notata*: JQ755806; *Oncopsis nigrofasciata:* KU056928).

### Sequence Assembly, Annotation, and Analysis

Genomes for the two species were sequenced using Illumina sequencing (Illumina HiSeq 2500 platform with 150 bp paired-end reads, average insert size of 350 bp and 2 GB clean data; Berry Genomic, Beijing, China) (*M. notata* BioSample accession: SAMN14542501; *O. nigrofasciata* BioSample accession: SAMN14542676). Using 600 bp COI sequences of *M. notata* (MT240255) and *O. nigrofasciata* (MT240256) as a reference, the sequences from the NGS data were mapped in Geneious v 2019.2.1 using the Map to reference function with a Medium-Low sensitivity and 5 times iteration. Then the previous results obtained were used as a new reference sequence, and the above assembly process was repeated until fishing out all the mitogenomic reads.

We first annotated the assembled sequences using the MITOS web server with the invertebrate genetic code (Bernt et al., 2013) and BLAST searches in NCBI (Johnson et al., 2008). The locations and secondary structures of 22 tRNAs were reconfirmed and predicted using tRNAscan-SE version 1.21 (Lowe and Eddy, 1997) and ARWEN version 1.2 (Laslett and Canbäck, 2008). The locations of two rRNA genes (*16S* rRNA and *12S* rRNA) were determined by comparing the homologous sequences with previously published mitochondrial sequences for the members of Hemiptera in GenBank. Secondary structures of rRNA genes were predicted based on previously reported models (Wang et al., 2017a, 2019a) variable regions of the elements were predicted using DNASIS version 2.5 (Hitachi Engineering, Tokyo, Japan) and RNA Structure (v 5.2) (Reuter and Mathews, 2010). We calculated strand asymmetry using the formulas: AT skew = (A − T)/(A + T) and GC skew = (G − C)/(G + C) (Perna and Kocher, 1995). Furthermore, base composition and codon usage of protein coding genes (PCGs) were analyzed using MEGA 7 (Kumar et al., 2016). The repeating units in mitogenomes were identified using the Tandem Repeats Finder tool (Benson, 1999).

### Sequence Alignment and Phylogenetic Analysis

Our phylogenetic analysis was based on 89 leafhoppers and five treehoppers as the ingroup. *Gaeana maculata* (KM244671) (Tang et al., 2014), *Magicicada tredecim* (NC041652) (Du Z. et al., 2019), and *Tettigades auropilosa* (KM000129) were selected as the members of the outgroup (Supplementary Table S2). Sequences of 13 PCGs and 2 rRNAs were used to infer the phylogenetic relationships within leafhoppers. Each of the PCGs (excluding stop codons) were initially aligned using MASCE v2 (Ranwez et al., 2018); gaps and ambiguous sites were removed using Gblocks 0.91b (Talavera and Castresana, 2007) with default settings. The rRNA genes were aligned with MAFFT v7 (Katoh and Standley, 2013) using the Q-INS-I strategy, and the poorly aligned positions and divergent regions were removed using Gblocks 0.91b under default settings (Talavera and Castresana, 2007). Alignments of individual genes were then concatenated as different datasets using MEGA 7 (Kumar et al., 2016).

The optimal partition scheme for each dataset and the best model for each partition was determined using PartitionFinder 2 under the AIC, and a greedy algorithm with linked branch lengths (Supplementary Tables S3–S5) (Lanfear et al., 2017). Phylogenetic trees were constructed using the Maximum likelihood (ML) method using IQ-TREE v1.6.12 (Lam-Tung et al., 2014), and Bayesian inference (BI) was performed using MrBayes 3.2.6 (Huelsenbeck and Ronquist, 2001) under the best schemes and models. ML estimation used an ultrafast bootstrap approximation approach with 10,000 replicates. BI analyses used default settings by simulating four independent runs for 100 million generations and sampling every 1,000 generations; after the average standard deviation of split frequencies fell below 0.001, the initial 25% of samples were discarded as burn-in and the remaining trees were used to calculate the posterior probabilities by generating a consensus tree.

## Results and Discussion

### Genome Organization and Composition

**FIGURE 1:**
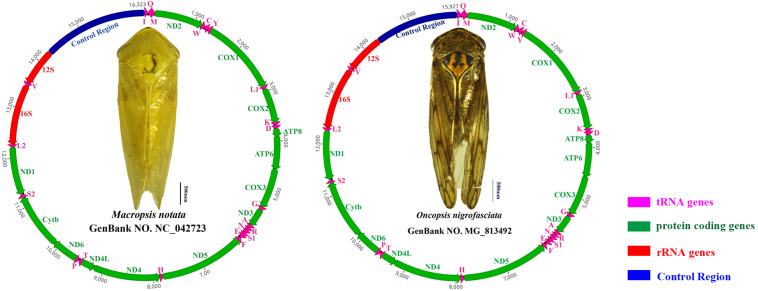
Circular maps of the mitochondrial genomes of *Macropsis notata* and *Oncopsis nigrofasciata*. Different colors indicate different types of genes; arrows indicate the direction of transcription for each gene.

The complete mitogenomes of *Macropsis notata* (NC042723) and *Oncopsis nigrofasciata* (MG813492) were assembled using their barcode sequences (COI fragments) as seeds to fish out the mitogenomic reads. The length of *M. notata* and *O. nigrofasciata* mitogenomes was 16,323 and 15,927 bp, respectively. Both contained 37 typical insect mitochondrial genes (13 PCGs, 22 tRNA genes, and 2 rRNA genes) and a long non-coding region (control region). The gene orders and arrangements of the two sequences are identical to those of most other leafhoppers (Figure 1; Du et al., 2017a; Liu et al., 2017; Choudhary et al., 2018; Wang et al., 2019a, b). Although the mitochondrial gene arrangement is relatively compact, not every gene is closely linked. In *M. notata*, a total of 32 bp overlaps were observed in 13 locations (from 1 to 7 bp), and intergenic spacers of 20 bp occur in seven locations (from 1 to 9 bp) (Supplementary Table S6). In *O. nigrofasciata*, a total of 71 bp overlaps were observed in 15 locations (from 1 to 10 bp), and intergenic spacers of 21 bp occur in seven locations (from 1 to 6 bp) (Supplementary Table S7).

The nucleotide compositions of the complete mitogenomes of *M. notata* and *O. nigrofasciata* were as follows (Table 1): (A) 44.3 and 44.4%; (T) 32.5 and 34.5%; (C) 13.2 and 11.4%; (G) 10 and 9.7%, respectively. The mitogenomes of both these species of Macropsinae exhibited a heavy AT nucleotide bias (76.8 and 79.0%), in accordance with the other leafhoppers mitogenomes (Du et al., 2017a, b; Mao et al., 2017; Choudhary et al., 2018; Dai et al., 2018; Wang et al., 2018). In addition, the GC content, AT skew, and GC skew were also calculated for the mitogenomes of *M. notata* and *O. nigrofasciata*, which showed a positive AT Skew (0.15 and 0.12) and negative GC Skew (–0.14 and –0.08).

**TABLE 1:** Skewed nucleotide composition of *Macropsis notata* and *Oncopsis nigrofasciata* mitogenomes.

Species name	Region	Total (bp)	A (%)	C (%)	G (%)	T (%)	A + T%	AT-skew	GC-skew
*Macropsis notata*	Whole genome	16323	44.3	13.2	10	32.5	76.8	0.15	–0.14
	PCGs	10989	32.4	13.2	12	42.1	74.5	–0.13	–0.05
	RNAs	3335	37.3	8.5	12.3	41.9	79.2	–0.06	0.18
	Control region	2068	48.8	6.8	7.4	37	85.8	0.14	0.04
*Oncopsis nigrofasciata*	Whole genome	15927	44.4	11.4	9.7	34.6	79	0.12	–0.08
	PCGs	10971	33.3	11.3	11.3	44.1	77.4	–0.14	0
	RNAs	3370	36.9	7.8	11.7	43.6	80.5	–0.08	0.2
	Control region	1616	44.6	6.3	7.6	41.5	86.1	0.04	0.09

### Protein-Coding Genes and Codon Usage

In total, 3,663 and 3,657 amino acids are encoded by the mitogenomes of *M. notata* and *O. nigrofasciata*, respectively. Among the 13 PCGs, the longest was *ND5* and the shortest was *ATP8*; four genes (*ND1, ND4, ND4L*, and *ND5*) were coded by the N-strand, whereas the other genes were coded by the J-strand (Supplementary Tables S6, S7). All PCGs started with ATN (ATA, ATT, ATC, ATG) and were terminated by TAA or TAG, except for ATP8, which started with TTG. This non-standard initial codon phenomenon is often observed in the mitochondrial genes of other leafhoppers, especially with ATP8 (Wang et al., 2018, 2019b; Yuan et al., 2019).

**FIGURE 2:**
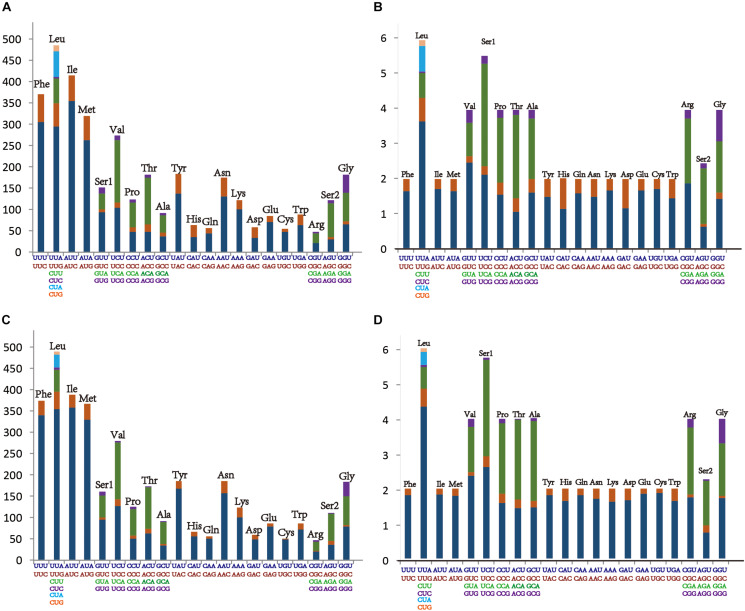
**(A)** Amino acid composition in *Macropsis notata*. **(B)** Relative synonymous codon usage in *Macropsis notata*. **(C)** Amino acid composition in *Oncopsis nigrofasciata*. **(D)** Relative synonymous codon usage in *Oncopsis nigrofasciata*. Codon families are indicated on the X-axis.

The average AT content of PCGs in the *M. notata* and *O. nigrofasciata* mitogenomes was 74.5 and 77.4%, with a slightly negative AT skew (–0.13 and –0.14) and GC skew (–0.05 and 0), respectively. The codon usage bias detected via the A + T content, the relative synonymous codon usage, and the amino acid composition in the PCGs of *M. notata* and *O. nigrofasciata* are presented in Figure 2 (except for the stop codons). The most frequently used amino acids were Leu, Ile, Phe, and Met, and each amino acid also preferred to use codons with a high AT content. The codon usage pattern of Macropsinae is thus highly consistent with that observed in previously sequenced mitogenomes of leafhoppers (Du et al., 2017a, b; Wang et al., 2017a, b).

### tRNAs and rRNAs

The secondary structures of tRNAs were typical clover-leaf structures, except for *trnS1* which formed a loop with the dihydrouridine (DHU) arm (Supplementary Figure S1), a common phenomenon in other insect mitogenomes (Cameron, 2014; Wang et al., 2015; Li et al., 2017; Du Y. et al., 2019). The length of the tRNAs ranged from 61 bp (*trnH*) to 68 bp (*trnM*) in *M. notata*, and from 62 bp (*trnV*) to 71 bp (*trnH*) in *O. nigrofasciata*. This difference is mainly caused by the loop region, specifically the variable loop.

*16S* rRNA genes were found between *trnV* and *trnL2*, and *12S* RNA genes were found between *trnV* and the control region. The length of the *16S* rRNA genes was 1,191 and 1,192 bp and that of the *12S* RNA genes was 733 and 748 bp in the *M. notata* and *O. nigrofasciata* mitogenomes, respectively. The secondary structure of rRNAs was predicted based on previously reported models (Supplementary Figures S2, S3). The rRNA gene sequences had highly conserved regions. Their secondary structures had structural similarities: six domains and 42 helices in *16S* rRNA genes, and three structural domains and 26 helices in *12S* rRNA genes were determined in both species. Moreover, there is little difference between the rRNA sequences of these species and those of previously predicted species (Wang et al., 2017a, b, 2018). Thus, these conserved structure units may provide some useful information for us to better understand the phylogenetic relationships within and among leafhoppers. Additionally, there is a need for studying more leafhopper rRNAs in future.

### Control Region

**FIGURE 3:**
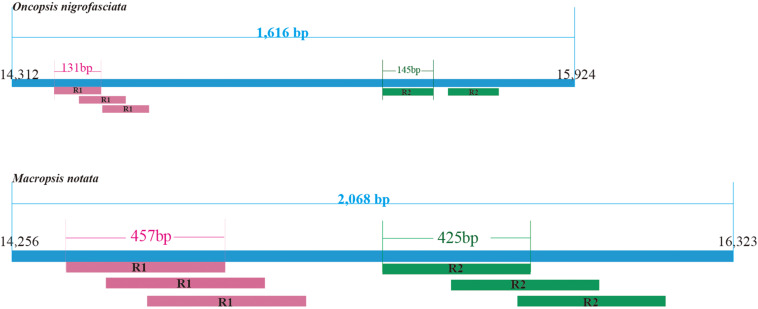
Structural organization of the control region in *Macropsis notata* and *Oncopsis nigrofasciata.*

Within the leafhoppers mitogenome, the control region has the largest variation in length and composition; it is the main cause of difference in mitogenome lengths. The stability control region is located between the genes *12S* RNA and *trnI*. It is 2,068 bp long with 85.8% AT content in *M. notata*, and 1,616 bp long with 86.1% AT content in *O. nigrofasciata*; in both cases, it contained various repeat sequences (Figure 3). Both *M. notata* and *O. nigrofasciata* contained two kinds of parallel repeat units. In *M. notata*, the first repeat unit (R1) was 457 bp long and the second repeat unit (R2) was 425 bp, both with three copies; in *O. nigrofasciata*, the first repeat unit (R1) was 131 bp long with three copies and the second repeat unit (R2) was 145 bp long with two copies. We did not find any relationship between the repeat units. Moreover, compared to the existing control region sequences, no obvious correlation or similarity was found.

### Phylogenetic Analyses

**FIGURE 4:**
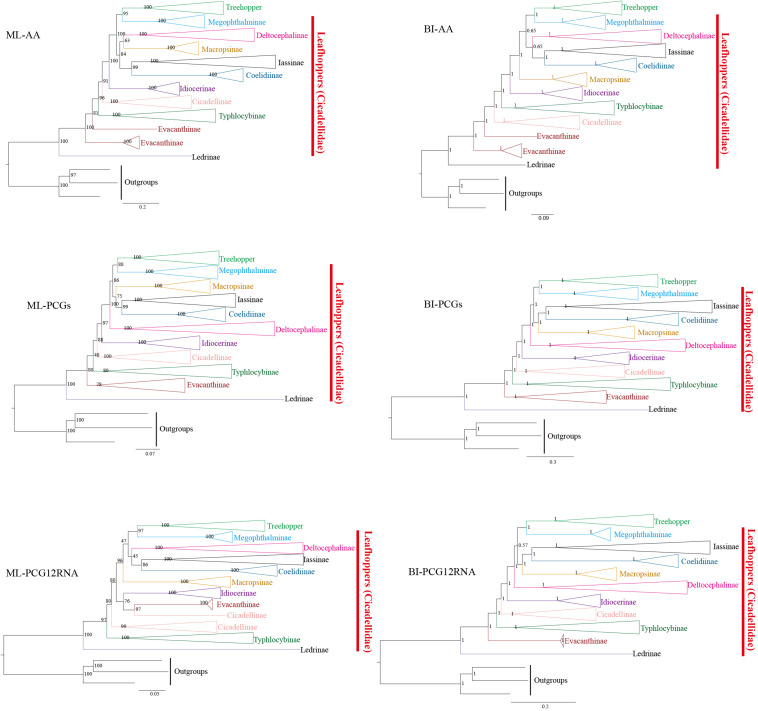
Phylogenetic trees of leafhoppers inferred by the maximum likelihood (ML) and Bayesian inference (BI) from different mitochondrial genomes datasets. AA: amino acid sequences of 13 PCGs from 97 species, PCGs: nucleotide sequences of 13 PCGs from 97 species, and PCG12RNA: the first and second codons of 13 PCGs and 2 rRNAs of 77 species.

ML and BI analyses were used to reconstruct the phylogenetic relationships among 10 subfamilies of leafhoppers, treehoppers, and three outgroups based on three datasets: (1) amino acid sequences of 13 PCGs from 97 species (AA, 3,500 amino acids), (2) nucleotide sequences of 13 PCGs from 97 species (PCGs, 10,506 bp), and (3) the first and second codons of 13 PCGs and 2 rRNAs of 77 species (PCG12RNA, 8,554 bp). Due to the unavailability of some mitogenome sequences, the number of species used in tree constructions varied. Based on three datasets, ML and BI analyses reconstructed six phylogenetic trees (ML-AA, ML- PCGs, ML-PCG12RNA, BI-AA, BI-PCGs, and BI-PCG12RNA), as shown in Figure 4 and Supplementary Figures S4–S9. The comparative analysis of the phylogenetic trees in this study indicated that some clade relationships were consistently recovered in the six trees even though the resulting topology was not exactly the same. Additionally, the most consistent phylogenetic relationships were seen in BI-PCGs and BI-PCG12RNA based on topology (Supplementary Figures S8, S9). In the present study, except for Evacanthinae and Cicadellinae, subfamilies Coelidiinae, Deltocephalinae, Iassinae, Idiocerinae, Ledrinae, Macropsinae, Megophthalminae, and Typhlocybinae have been reconstructed into monophyletic groups with strong support in all analyses [Bootstrap support values (BS) = 100, Bayesian posterior probability (PP) = 1] (Figure 4). In this study, some relationships are very stable. For example, Iassinae emerged as the sister group to Coelidiinae; treehoppers formed one clade and exhibited a sister relationship with Megophthalminae. This result supported that treehoppers were derived from paraphyletic Cicadellidae, which has been proven in previous studies (Dietrich et al., 2001, 2017; Zhao and Liang, 2016; Du et al., 2017a,b; Du Y. et al. 2019; Skinner et al., 2019). As previously reported by Wang et al. (2019a) Ledrinae was found to be the sister group of all leafhoppers and treehoppers present in our trees. Unfortunately, the relationships of Macropsinae have not been fully resolved in this study. With regard to ML-PCGs, BI-PCGs, and BI-PCG12RNA, Macropsinae is the sister group to Iassinae and Coelidiinae. With regard to ML-AA, Macropsinae is the sister group to Deltocephalinae;. With regard to BI-AA and ML-PCG12RNA, Macropsinae is sister group to {[(Iassinae, Coelidiinae), Deltocephalinae] (Treehopper, Megophthalminae)}.

There are a few discrepancies in this study when compared to the results of previous phylogenetic studies of members from Cicadellidae. Although all Deltocephalinae species are grouped into one clade, the relationships between Deltocephalinae and other subfamilies of different trees are not strongly supported. Deltocephalinae is the sister group for (treehopper + Megophthalminae) + [(Iassinae + Coelidinae) + Macropsinae] with regard to the BI-PCGs and BI-PCG12RNA (Supplementary Figures S8, S9). With regard to BI-AA, Evacanthinae was reconstituted into a polyphyletic group (Supplementary Figure S7) (PP = 1), and in ML-PCG12RNA, Cicadellinae was reconstituted into a polyphyletic group. Some species were recovered with Evacanthinae (Supplementary Figure S6) (BS = 97). This may be due to a too sparse taxon sampling. In this study, BI trees are more congruent and better supported than ML trees. In BI, the results of PCGs and PCG12RNA are identical. The difference between AA and PCGs may have arisen due to the fact that some evolutionary information is lost when nucleotides are translated into amino acids. Further sampling from more taxonomic samples and molecular data will elucidate the unclear relationships between these subfamilies and provide a better understanding of the phylogenetic and evolutionary relationships among Membracoidea.

## Conclusion

In the present study, we report similarities between the mitogenomes of *Macropsis notata* and *Oncopsis nigrofasciata* in Macropsinae. These form the first and second available mitogenomes for Macropsinae. The length of *M. notata* and *O. nigrofasciata* mitogenomes was 16,323 and 15,927 bp, respectively. This variation in length is mainly caused by the control region; leafhoppers mitogenome lengths are usually between 15,131 bp (*Trocnadella arisana*) and 16,811 bp (*Parocerus laurifoliae*). Both mitogenomes contained 37 typical insect mitochondrial genes and a control region. Except for the control region, there are no long non-coding sequences (1–9 bp). Thus, their genes are very compact, there is no rearrangement, and these two sequences have a high percentage of identical sites when aligned. Within the control region, both mitogenomes contained two kinds of parallel repeat units.

The results of phylogenetic analyses indicate that leafhoppers are paraphyletic with respect to treehoppers, and that most subfamilies are monophyletic groups within leafhoppers. Unfortunately, the phylogenetic position of the Macropsinae is not stable. With regard to ML-PCGs, BI-PCGs, and BI-PCG12RNA, Macropsinae is the sister group with Iassinae and Coelidiinae; with regard to ML-AA, Macropsinae is a sister group to Deltocephalinae; and with regard to BI-AA and ML-PCG12RNA, Macropsinae is the sister group to {[(Iassinae, Coelidiinae), Deltocephalinae] (Treehoppers, Megophthalminae)}. Interestingly, in all the analyses, Deltocephalinae does not represent the sister group of other leafhoppers species. In this study, we simply elucidated the characteristics of the mitochondrial genome of Cicadellidae, and preliminarily discussed the phylogenetic relationships among members of Cicadellidae based on the existing mitochondrial genome data. It is possible that the due to limited samples and sequences, Cicadellinae and Evacanthinae were reconstituted into a polyphyletic group. We hope that subsequent research can help us prove these relationships among Cicadellidae.

## Data Availability Statement

Publicly available datasets were analyzed in this study. These data can be found here: GenBank Accession Nos. NC042723 and MG813492.

## Author Contributions

R-HD and M-FY conceived and designed the study, and critically revised the manuscript. J-JW and Y-FW performed the experiments and analyzed the data. J-JW drafted the manuscript. R-HD provided financial support.

## Conflict of Interest

The authors declare that the research was conducted in the absence of any commercial or financial relationships that could be construed as a potential conflict of interest.

## Footnotes

1 www.ncbi.nlm.nih.gov/blast/blast.cgi
