# Supplementary material for: The Phylogenetic Implications of the Mitochondrial Genomes of Macropsis notata and Oncopsis nigrofasciata
Source: Front Genet. 2020 May 20;11:443. doi: 10.3389/fgene.2020.00443 (PMC7251781; doi:10.3389/fgene.2020.00443)
Supplement: Supplementary file 1 [file Data_Sheet_1.docx]

**Supplementary Figures**

The phylogenetic implications of the mitochondrial genomes of *Macropsis notata* and *Oncopsis nigrofasciata*

Jiajia Wang, Yunfei Wu, Maofa Yang, Renhuai Dai*

Institute of Entomology, Guizhou University, The Provincial Key Laboratory for Agricultural Pest Management Mountainous Region, Guiyang, Guizhou 550025, People’s Republic of China

*** Correspondence:**Renhuai Dai

Email: [dmolbio@126.com](mailto:dmolbio@126.com)

Keywords: leafhopper, *Macropsis notata*, *Oncopsis nigrofasciata*, mitogenome, phylogenetic analyses


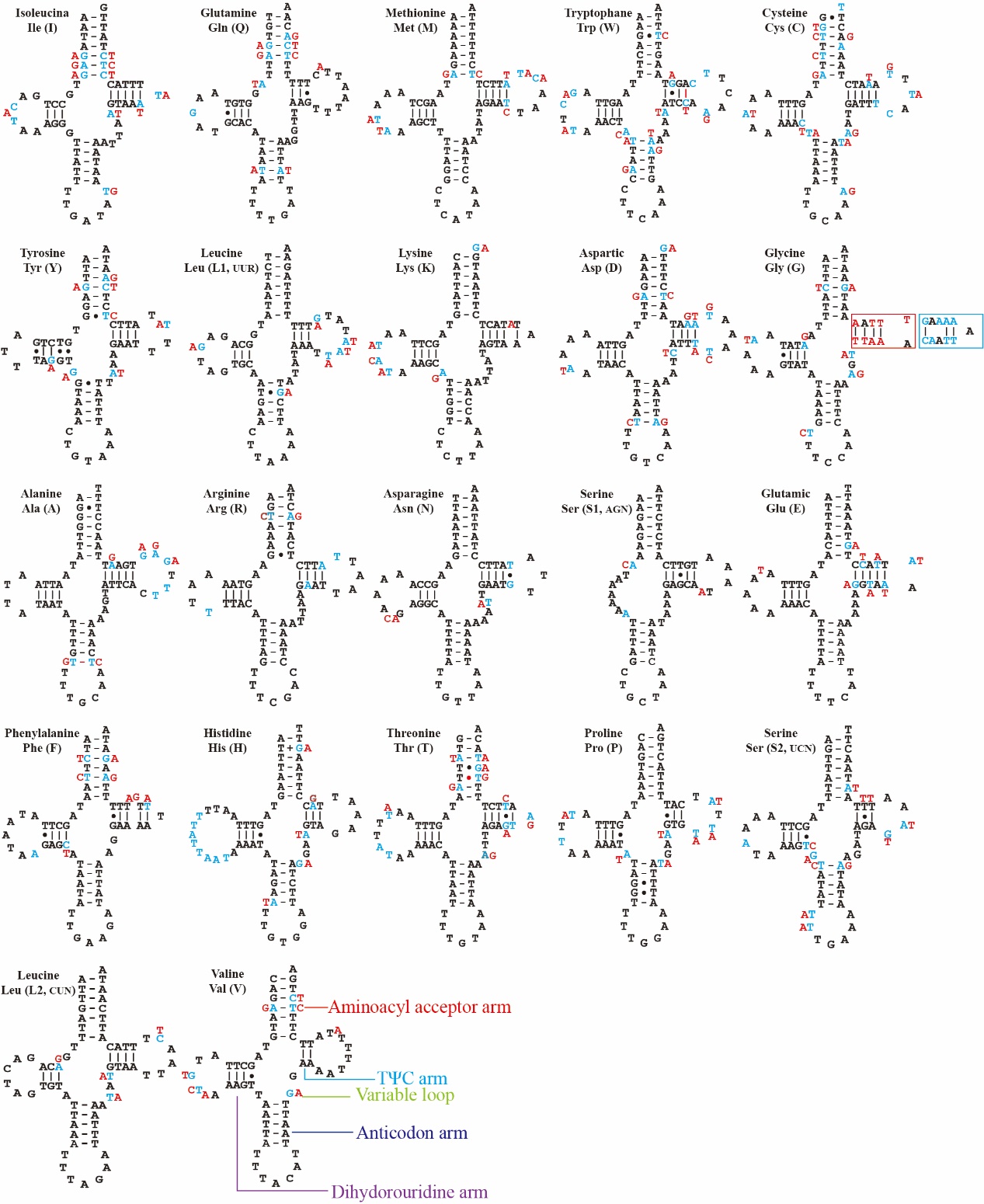


Figure S1 Predicted secondary structures of tRNAs in *Macropsis notata* and *Oncopsis nigrofasciata*. Dashes (−) indicate Watson–Crick base pairing and dots (•) indicate G–U base pairing. Blue (*Macropsis notata*) and red (*Oncopsis nigrofasciata*) indicate different nucleotides in the position.


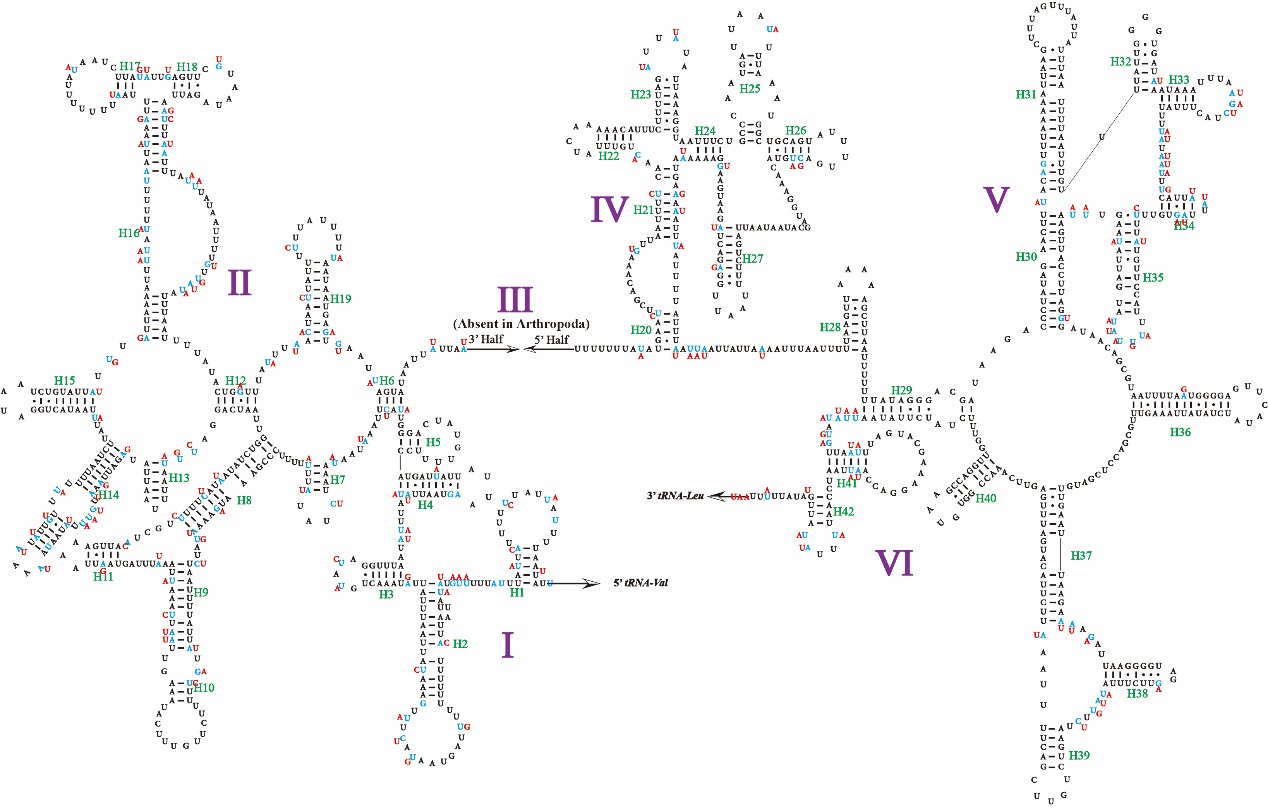
Figure S2 Predicted secondary structures of 16S rRNAs in *Macropsis notata* and *Oncopsis nigrofasciata*. Dashes (−) indicate Watson–Crick base pairing and dots (•) indicate G–U base pairing. Blue (*Macropsis notata*) and red (*Oncopsis nigrofasciata*) indicate different nucleotides in the position.


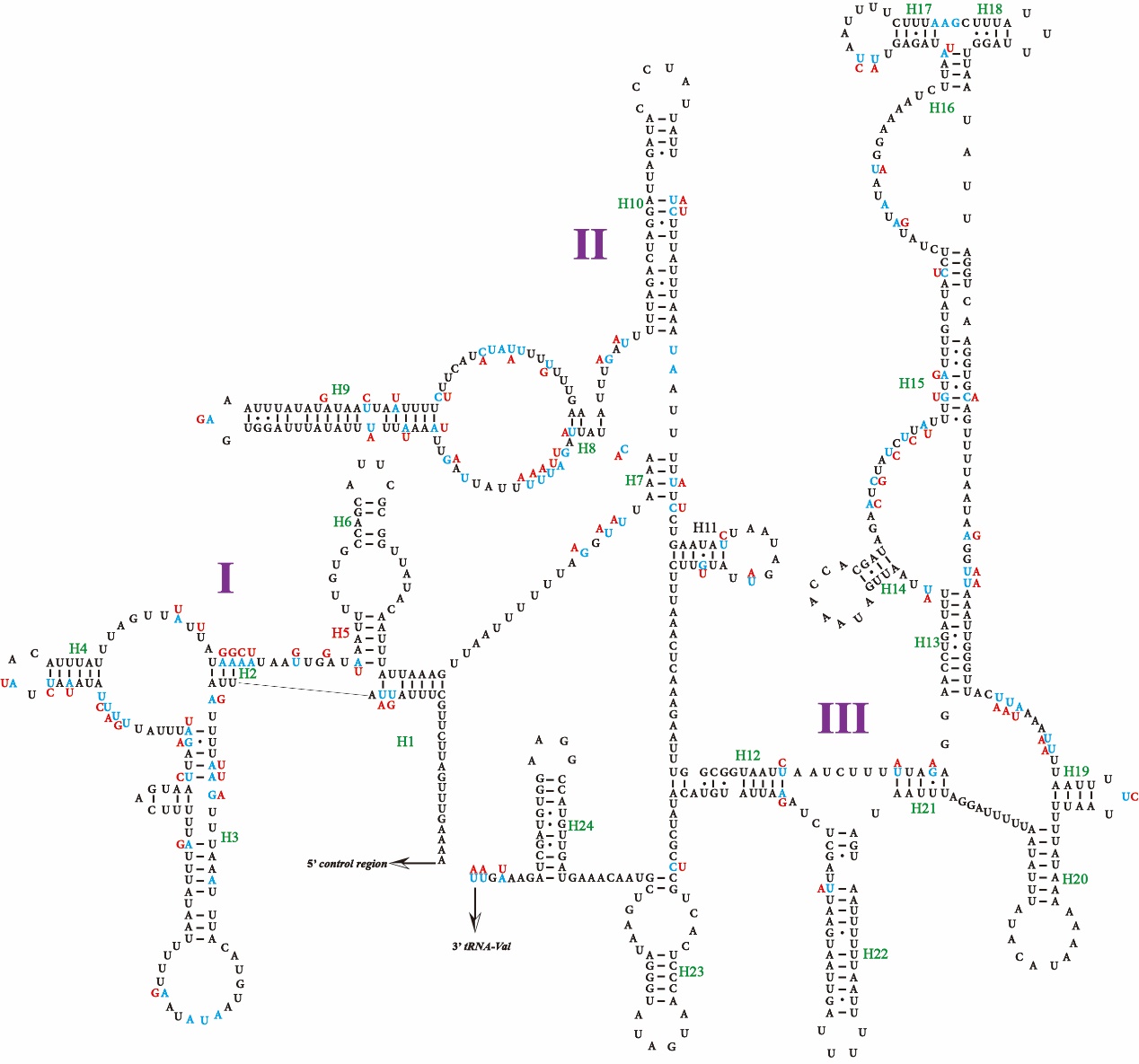


Figure S3 Predicted secondary structures of the 12S rRNAs in *Macropsis notata* and *Oncopsis nigrofasciata*. Dashes (−) indicate Watson–Crick base pairing and dots (•) indicate G–U base pairing. Blue (*Macropsis notata*) and red (*Oncopsis nigrofasciata*) indicate different nucleotides in the position.


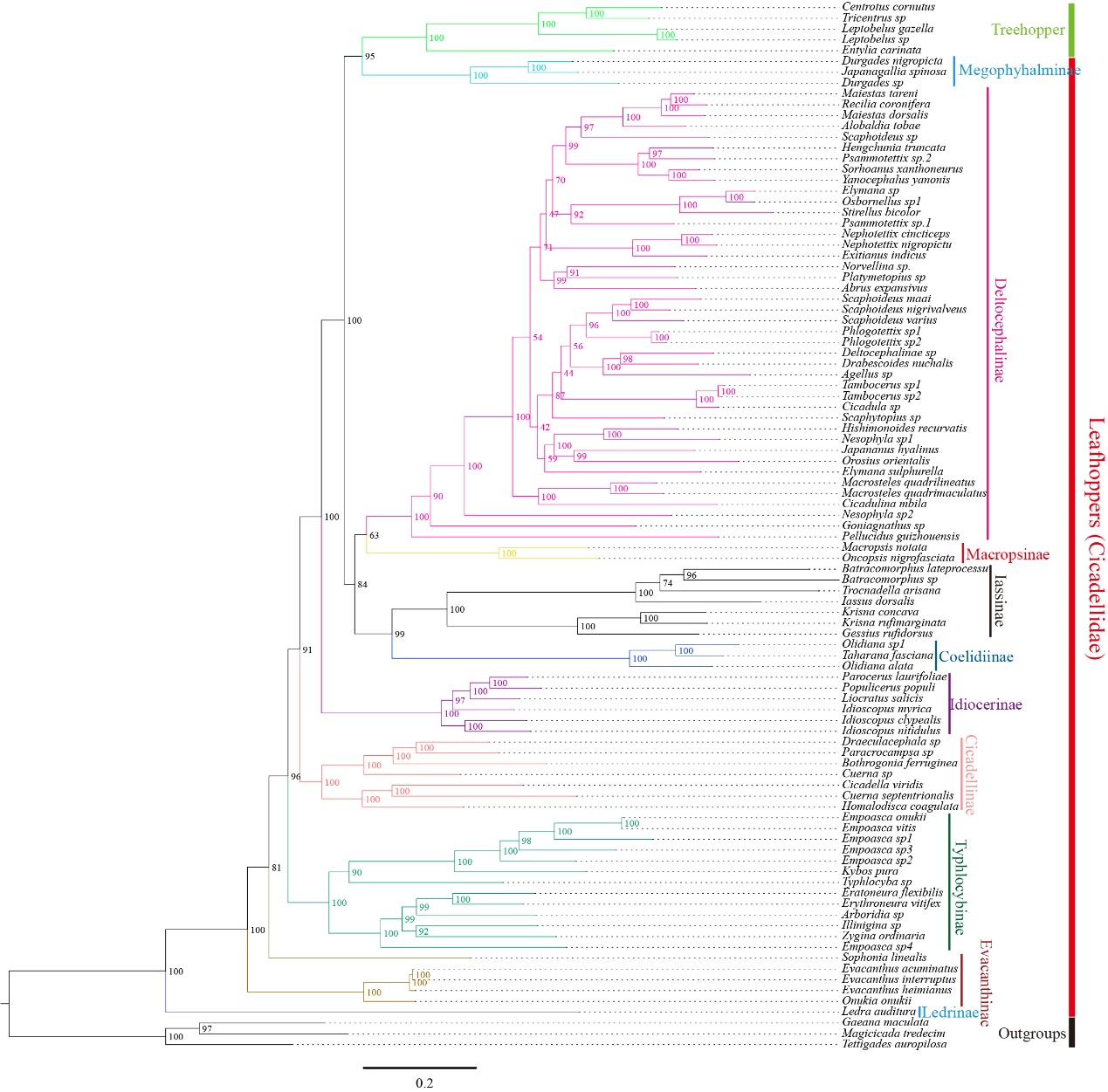


Figure S4 Phylogenetic tree of leafhoppers inferred by the maximum likelihood based on amino acid sequences of 13 PCGs.


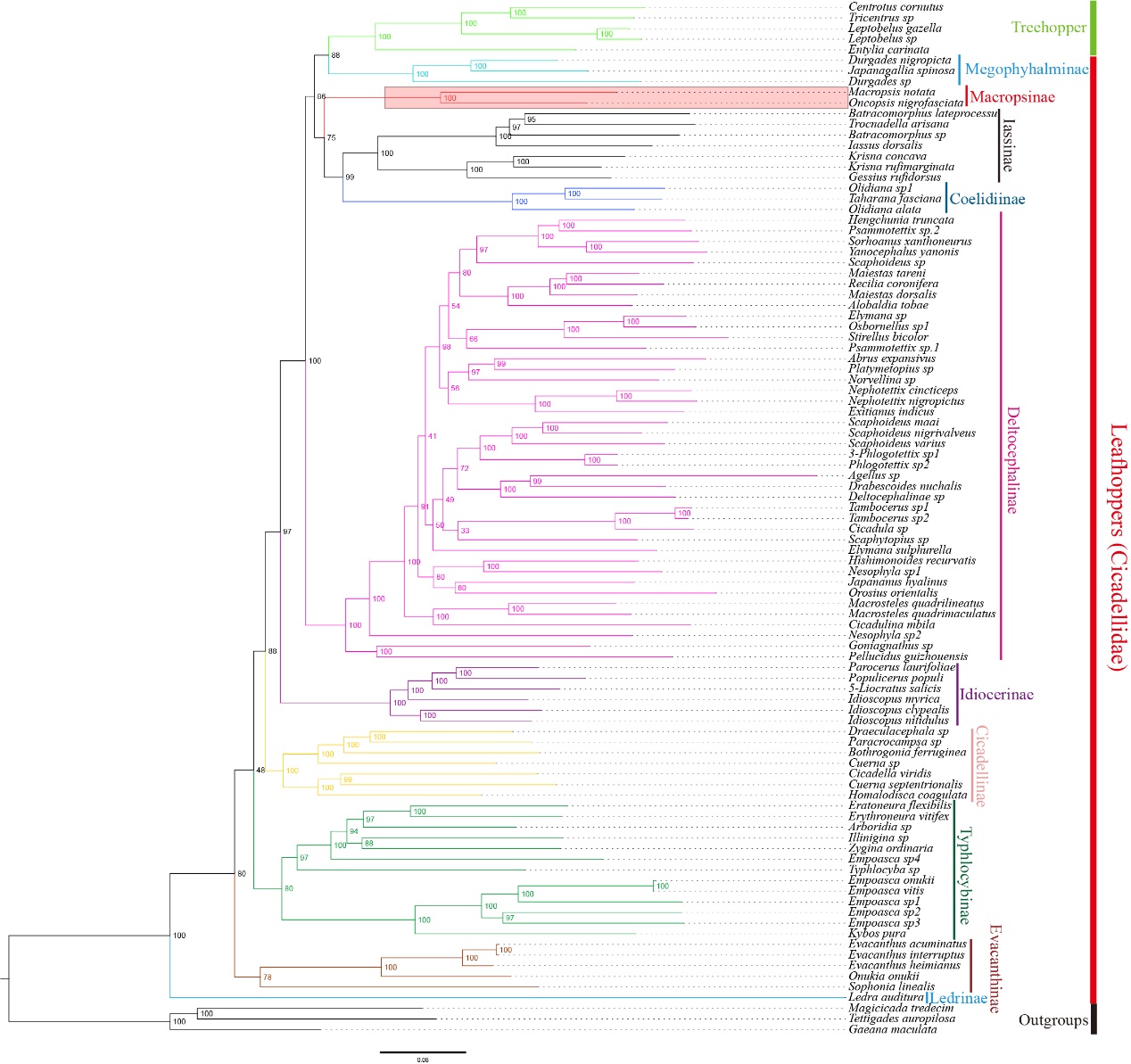


Figure S5 Phylogenetic tree of leafhoppers inferred by the maximum likelihood based on nucleotide sequences of 13 PCGs.


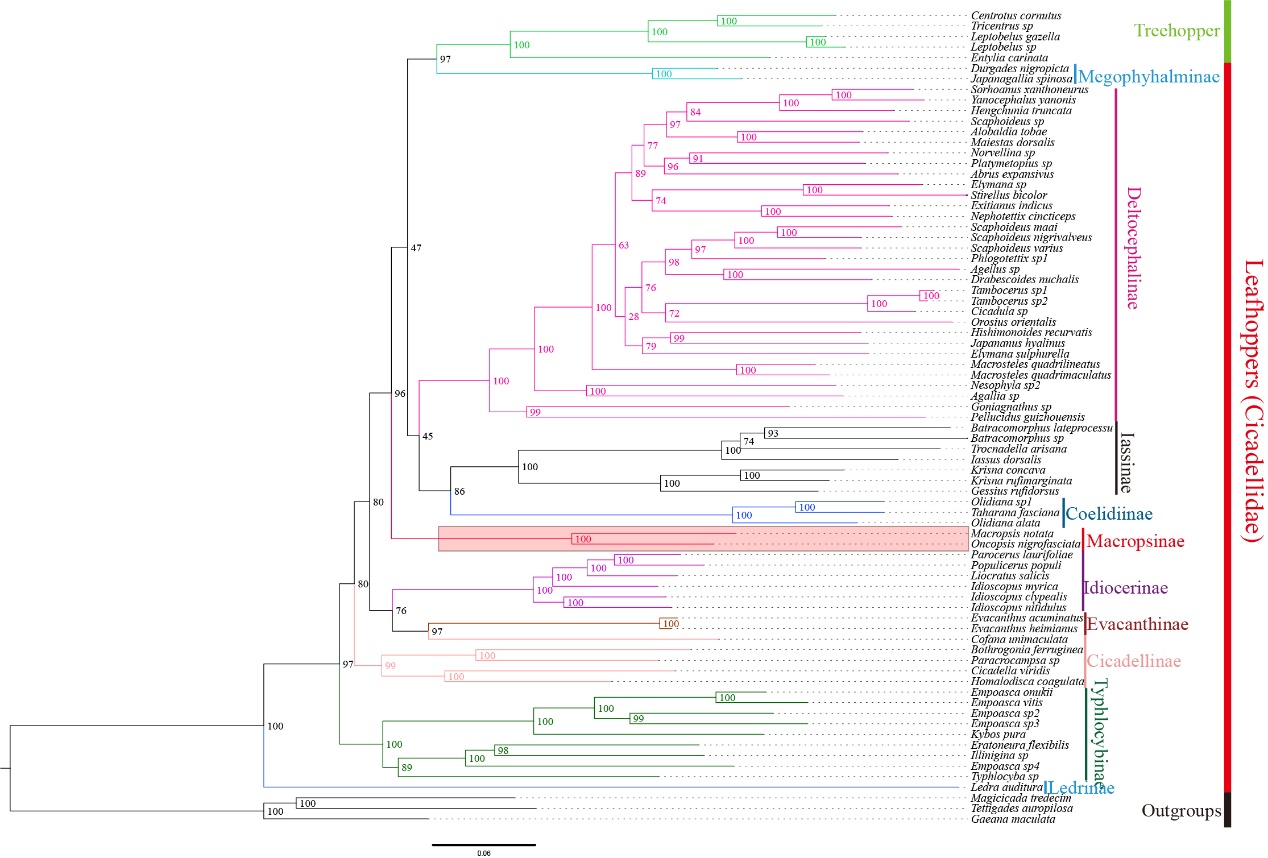


Figure S6 Phylogenetic tree of leafhoppers inferred by the maximum likelihood based on nucleotides of the first and second codons of 13 PCs from 13 PCGs and 2 rRNAs.


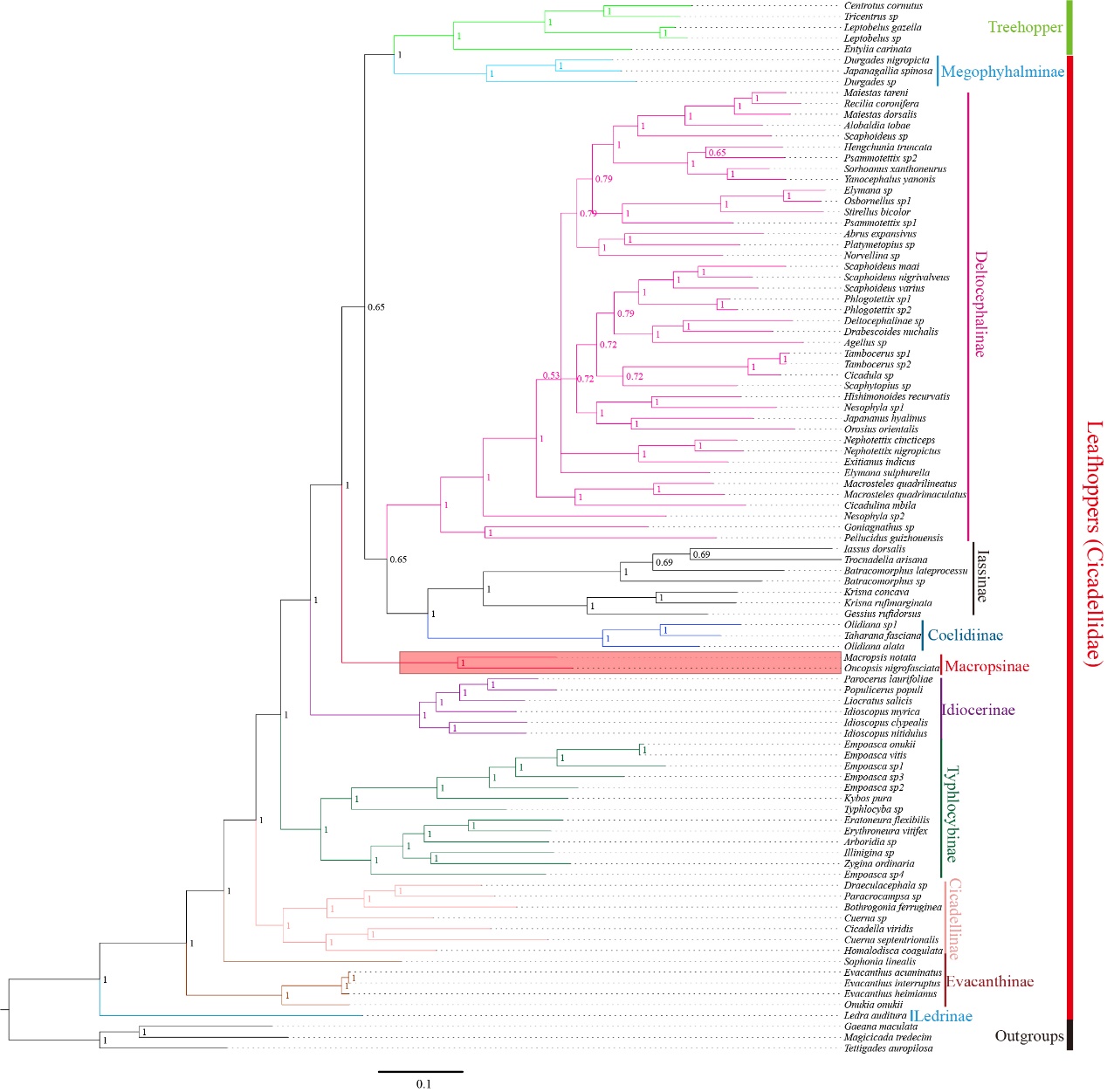


Figure S7 Phylogenetic tree of leafhoppers inferred by Bayesian inference based on amino acid sequences of 13 PCGs.


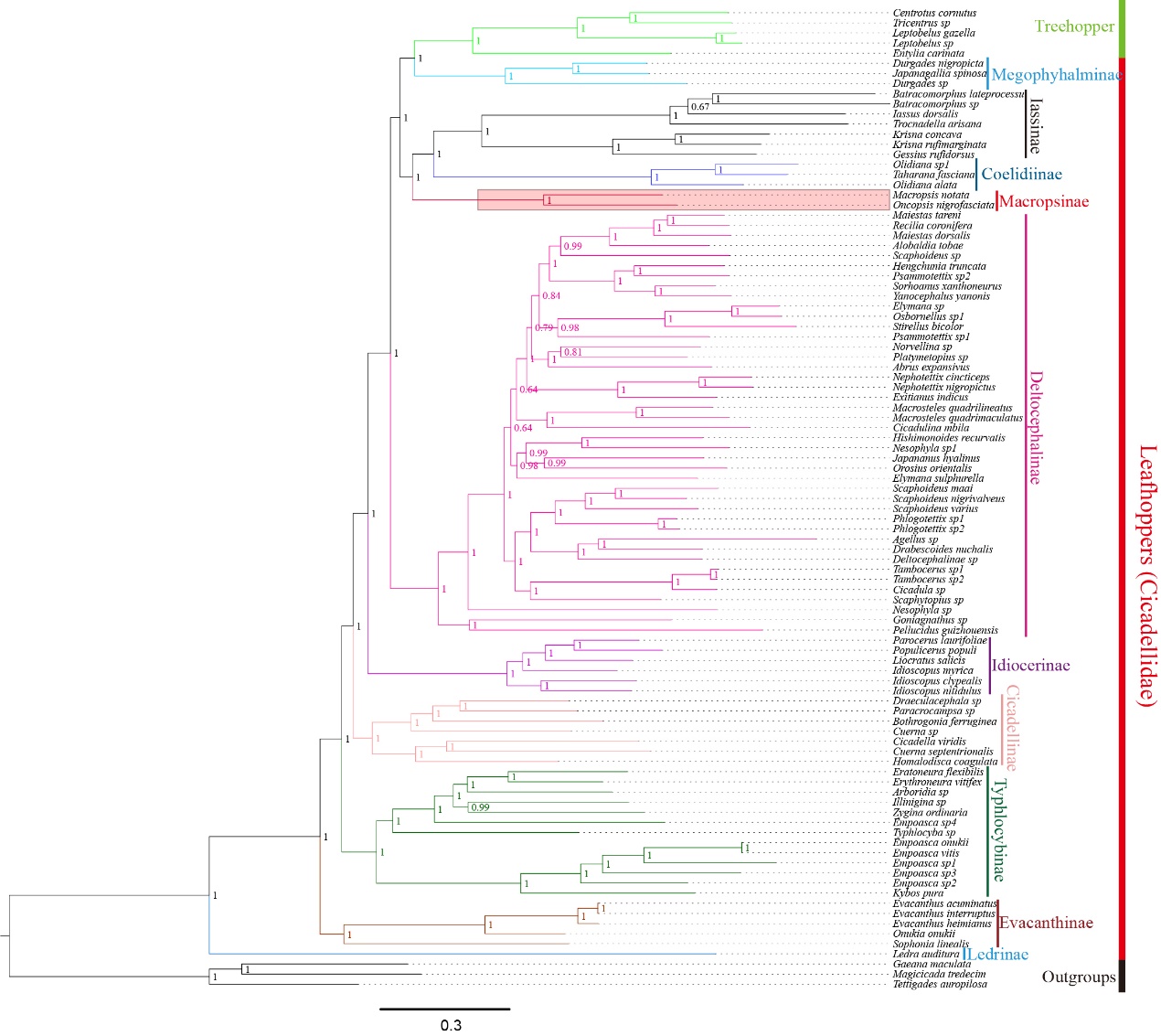


Figure S8 Phylogenetic tree of leafhoppers inferred by Bayesian inference based on nucleotide sequences of 13 PCGs.


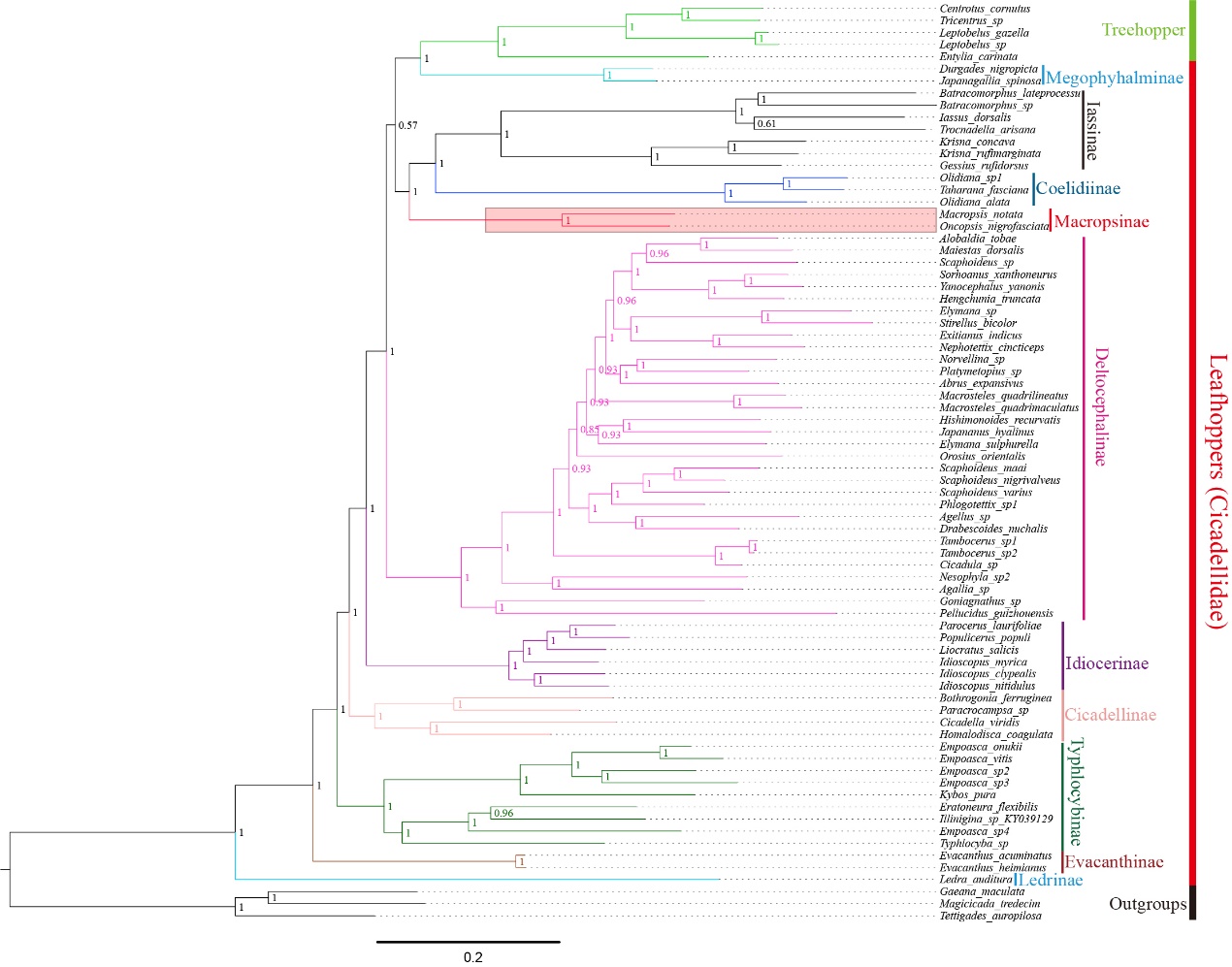


Figure S9 Phylogenetic tree of leafhoppers inferred by Bayesian inference based on nucleotides of the first and second codons of 13 PCs from 13 PCGs and 2 rRNAs.
