# Supplementary material for: The Phylogenetic Implications of the Mitochondrial Genomes of Macropsis notata and Oncopsis nigrofasciata
Source: Front Genet. 2020 May 20;11:443. doi: 10.3389/fgene.2020.00443 (PMC7251781; doi:10.3389/fgene.2020.00443)
Supplement: Supplementary file 2 [file Data_Sheet_2.docx]

**Supplementary Tables**

The phylogenetic implications of the mitochondrial genomes of *Macropsis notata* and *Oncopsis nigrofasciata*

Jiajia Wang, Yunfei Wu, Maofa Yang, Renhuai Dai*

Institute of Entomology, Guizhou University, The Provincial Key Laboratory for Agricultural Pest Management Mountainous Region, Guiyang, Guizhou 550025, People’s Republic of China

*** Correspondence:**Renhuai Dai

Email: [dmolbio@126.com](mailto:dmolbio@126.com)

Keywords: leafhopper, *Macropsis notata*, *Oncopsis nigrofasciata*, mitogenome, phylogenetic analyses

**Table S1. Collection information of specimen in the present study.**

| **Name** | **Locality** | **Storage number** | **Time** |
| --- | --- | --- | --- |
| *M. notata* | - 106°41′8″E, 26°26′58″N | GZU-HO-000006 | May 2, 2016 |
| *O. nigrofasciata* | - 106°18′16″E, 35°39′59″N | GZU-HO-000107 | August 9, 2016 |

Note: *M. notata* indicates *Macropsis notata* and *O. nigrofasciata* indicates *Oncopsis nigrofasciata.*

**Table S2 Mitochondrial genomes used in this study**

| **Subfamily** | **Species name** | **GenBank No.** | **Reference** |
| --- | --- | --- | --- |
| **Cicadellinae** | *Bothrogonia ferruginea* | KU167550 | Unpublished |
|  | *Cicadella viridis* | KY752061 | Unpublished |
|  | *Cofana unimaculata* | MK251095 | (Song et al., 2019) |
|  | *Cuerna septentrionalis* | MK251107 | (Song et al., 2019) |
|  | *Cuerna* sp. | KX437741 | (Song et al., 2018) |
|  | *Draeculacephala* sp. | MK251110 | (Song et al., 2019) |
|  | *Eratoneura flexibilis* | MK251094 | (Song et al., 2019) |
|  | *Homalodisca coagulata* | AY875213 | Unpublished |
|  | *Paracrocampsa* sp. | MK251085 | (Song et al., 2019) |
| **Coelidiinae** | *Taharana fasciana* | NC_036015 | (Wang et al., 2017b) |
|  | *Olidiana* sp.1 | KY039119 | (Song et al., 2019) |
|  | *Olidiana alata* | MK251096 | (Song et al., 2019) |
| **Deltocephalinae** | *Abrus expansivus* | MK033020 | (Wang and Xing, 2019) |
|  | *Agellus* sp. | KX437738 | (Song et al., 2018) |
|  | *Alobaldia tobae* | KY039116 | Unpublished |
|  | *Cicadula* sp*.* | KX437724 | (Song et al., 2018) |
|  | *Cicadulina mbila* | MK251127 | (Song et al., 2019) |
|  | *Deltocephalinae* sp. | KX437726 | (Song et al., 2018) |
|  | *Dryadomorpha* sp. | KX437736 | (Song et al., 2018) |
|  | *Drabescoides nuchalis* | NC_028154 | (Wu et al., 2016) |
|  | *Elymana* sp. | MK251130 | (Song et al., 2019) |
|  | *Elymana sulphurella* | MK251106 | (Song et al., 2019) |
|  | *Exitianus indicus* | KY039128 | (Song et al., 2017) |
|  | *Goniagnathus* sp. | MK251086 | (Song et al., 2019) |
|  | *Hengchunia truncata* | MK251146 | (Song et al., 2019) |
|  | *Hishimonoides recurvatis* | KY364883 | (Song et al., 2018) |
|  | *Japananus hyalinus* | NC_036298 | (Du et al., 2017b) |
|  | *Macrosteles quadrilineatus* | NC_034781 | (Mao et al., 2017) |
|  | *Macrosteles quadrimaculatus* | NC_039560 | (Du et al., 2019a) |
|  | *Maiestas dorsalis* | NC_036296 | (Du et al., 2017b) |
|  | *Maiestas tareni* | MK251134 | (Song et al., 2019) |
|  | *Nephotettix cincticeps* | NC_026977 | (Song et al., 2017) |
|  | *Nephotettix nigropictus* | MK251135 | (Song et al., 2019) |
|  | *Nesophyla* sp.1 | MK251138 | (Song et al., 2019) |
|  | *Nesophyla* sp.2 | MK251087 | (Song et al., 2019) |
|  | *Norvellina* sp. | KY039131 | (Song et al., 2017) |
|  | *Orosius orientalis* | KY039146 | (Song et al., 2017) |
|  | *Osbornellus* sp.1 | MK251136 | (Song et al., 2018) |
|  | *Pellucidus guizhouensis* | MF784429 | Unpublished |
|  | *Phlogotettix* sp.1 | KY039135 | (Song et al., 2017) |
|  | *Phlogotettix* sp.2 | KX437721 | (Song et al., 2017) |
|  | *Platymetopius* sp. | MK251121 | (Song et al., 2019) |
|  | *Psammotettix* sp.1 | KX437725 | (Song et al., 2019) |
|  | *Psammotettix* sp.2 | KX437742 | (Song et al., 2019) |
|  | *Recilia coronifera* | MK251142 | (Song et al., 2019) |
|  | *Scaphoideus maai* | KY817243 | (Du et al., 2017a) |
|  | *Scaphoideus nigrivalveus* | KY817244 | (Du et al., 2017a) |
|  | *Scaphoideus* sp. | MK251143 | (Song et al., 2019) |
|  | *Scaphoideus varius* | KY817245 | (Du et al., 2017a) |
|  | *Scaphytopius* sp. | MK251144 | (Song et al., 2019) |
|  | *Sorhoanus xanthoneurus* | MK251116 | (Song et al., 2019) |
|  | *Stirellus bicolor* | MK251122 | (Song et al., 2019) |
|  | *Tambocerus* sp.1 | MK251123 | (Yu et al., 2017) |
|  | *Tambocerus* sp.2 | KT827824 | (Song et al., 2019) |
|  | *Yanocephalus yanonis* | NC_036131 | (Song et al., 2017) |
| **Iassinae** | *Batracomorphus* sp. | MK251097 | (Song et al., 2019) |
|  | *Batracomorphus lateprocessu* | MG813489 | Unpublished |
|  | *Gessius rufidorsus* | MN577633 |  |
|  | *Iassus dorsalis* | MN577634 |  |
|  | *Krisna concava* | MN577635 |  |
|  | *Krisna rufimarginata* | MN577636 |  |
|  | *Trocnadella arisana* | NC_036480 |  |
| **Idiocerinae** | *Idiocerus laurifoliae* | NC_039741 | (Wang et al., 2018) |
|  | *Idioscopus clypealis* | NC_039642 | (Dai et al., 2018) |
|  | *Idioscopus myrica* | MH492317 | (Wang et al., 2018) |
|  | *Idioscopus nitidulus* | NC_029203 | (Choudhary et al., 2018) |
|  | *Liocratus salicis* | MG813490 | (Wang et al., 2018) |
|  | *Populicerus populi* | NC_039427 | (Wang et al., 2018) |
| **Macropsinae** | *Macropsis notata* | NC_042723 | This study |
|  | *Oncopsis nigrofasciata* | MG_813492 |  |
| **Megophthalminae** | *Japanagallia spinosa* | NC_035685.1 | (Wang et al., 2017a) |
|  | *Durgades nigropicta* | NC_035684.1 | (Wang et al., 2017a) |
|  | *Agallia* sp. | MK251084 | (Song et al., 2019) |
|  | *Durgades* sp. | MK251102 | (Song et al., 2019) |
| **Typhlocybinae** | *Arboridia* sp. | MK251098 | (Song et al., 2019) |
|  | *Empoasca onukii* | NC_037210.1 | Unpublished |
|  | *Empoasca* sp.1 | MK251099 | (Song et al., 2019) |
|  | *Empoasca* sp.2 | MK251093 | (Song et al., 2019) |
|  | *Empoasca* sp.3 | MK251111 | (Song et al., 2019) |
|  | *Empoasca* sp.4 | KX437737.1 | (Song et al., 2018) |
|  | *Empoasca vitis* | NC_024838.1 | (Zhou et al., 2016) |
|  | *Eratoneura flexibilis* | MK251094 | (Song et al., 2019) |
|  | *Erythroneura vitifex* | MK251100 | (Song et al., 2019) |
|  | *Illinigina* sp. | KY039129.1 | (Song et al., 2019) |
|  | *Kybos pura* | MK251088 | (Song et al., 2019) |
|  | *Typhlocyba* sp. | KY039138.1 | (Song et al., 2019) |
|  | *Zygina ordinaria* | MK251104 | (Song et al., 2019) |
| **Evacanthinae** | *Evacanthus heimianus* | MG813486 | (Wang et al., 2019) |
|  | *Evacanthus interruptus* | MK251114 | (Song et al., 2019) |
|  | *Onukia onukii* | MK251119 | (Song et al., 2019) |
|  | *Sophonia linealis* | KX437723.1 | (Song et al., 2018) |
| **Ledrinae** | *Ledra auditura* | MK387845 | Unpublished |
| **Treehopper** | *Centrotus cornutus* | KX437728 | (Song et al., 2018) |
|  | *Entylia_carinata* | NC_033539 | (Mao et al., 2016) |
|  | *Leptobelus_gazella* | NC_023219 | (Zhao and Liang, 2016) |
|  | *Leptobelus*_sp. | JQ910984 | (Li et al., 2017) |
|  | *Tricentrus_*sp. | KY039118 | Unpublished |
| **Outgroups** | *Gaeana maculata* | KM244671 | (Tang et al., 2014) |
|  | *Magicicada tredecim* | NC_041652 | (Du et al., 2019b) |
|  | *Tettigades auropilosa* | KM000129 | Unpublished |

**References**

Choudhary, J.S., Naaz, N., Das, B., Bhatt, B.P., and Prabhakar, C.S. (2018). Complete mitochondrial genome of Idioscopus nitidulus (Hemiptera: Cicadellidae). *Mitochondrial DNA Part B* 3，2018**,** 191192.

Dai, R.H., Wang, J.J., and Yang, M.F. (2018). The complete mitochondrial genome of the leafhopper Idioscopus clypealis (Hemiptera: Cicadellidae: Idiocerinae). *Mitochondrial DNA Part B* 3**,** 32-33

Du, Y., Dai, W., and Dietrich, C.H. (2017a). Mitochondrial genomic variation and hylogenetic relationships of three groups in the genus Scaphoideus (Hemiptera: Cicadellidae: Deltocephalinae). *Scientific Reports* 7.

Du, Y., Dietrich, C.H., and Dai, W. (2019a). Complete mitochondrial genome of Macrosteles quadrimaculatus (Matsumura) (Hemiptera: Cicadellidae: Deltocephalinae) with a shared tRNA rearrangement and its phylogenetic implications. *International journal of biological macromolecules* 122**,** 1027-1034.

Du, Y., Zhang, C., Dietrich, C.H., Zhang, Y., and Dai, W. (2017b). Characterization of the complete mitochondrial genomes of Maiestas dorsalis and Japananus hyalinus (Hemiptera: Cicadellidae) and comparison with other Membracoidea. *Scientific Reports* 7.

Du, Z., Hasegawa, H., Cooley, J.R., Simon, C., Yoshimura, J., Cai, W., Sota, T., Li, H.J.M.B., and Evolution (2019b). Mitochondrial Genomics Reveals Shared Phylogeographic Patterns and Demographic History among Three Periodical Cicada Species Groups. 36**,** 1187-1200.

Li, H., Leavengood, J.M., Chapman, E.G., Burkhardt, D., Song, F., Jiang, P., Liu, J.P., Zhou, X.G., and Cai, W.Z. (2017). Mitochondrial phylogenomics of Hemiptera reveals adaptive innovations driving the diversification of true bugs. *Proceedings of the Royal Society B-Biological Sciences* 284.

Mao, M., Yang, X., and Bennett, G. (2016). The complete mitochondrial genome of Entylia carinata (Hemiptera: Membracidae). *Mitochondrial DNA Part B* 1**,** 662-663.

Mao, M., Yang, X.S., and Bennett, G. (2017). The complete mitochondrial genome of Macrosteles quadrilineatus (Hemiptera: Cicadellidae). *Mitochondrial DNA Part B-Resources* 2**,** 173-175.

Song, N., Cai, W., and Li, H. (2018). Insufficient power of mitogenomic data in resolving the auchenorrhynchan monophyly. *Zoological Journal of the Linnean Society* 183**,** 776-790.

Song, N., Cai, W.Z., and Li, H. (2017). Deep-level phylogeny of Cicadomorpha inferred from mitochondrial genomes sequenced by NGS. *Scientific Reports* 7.

Song, N., Zhang, H., and Zhao, T. (2019). Insights into the phylogeny of Hemiptera from increased mitogenomic taxon sampling. *Molecular Phylogenetics and Evolution* 137**,** 236-249.

Tang, M., Tan, M., Meng, G., Yang, S., Su, X., Liu, S., Song, W., Li, Y., Wu, Q., and Zhang, A.J.N.a.R. (2014). Multiplex sequencing of pooled mitochondrial genomes—a crucial step toward biodiversity analysis using mito-metagenomics. 42**,** e166-e166.

Wang, J.-J., and Xing, J.-C. (2019). Complete mitochondrial genome of Abrus expansivus (Hemiptera: Cicadellidae: Deltocephalinae) from China. *Mitochondrial DNA Part B-Resources* 4**,** 197-198.

Wang, J.J., Yang, M.-F., Dai, R.-H., and Li, H. (2019). Complete mitochondrial genome of Evacanthus heimianus (Hemiptera: Cicadellidae: Evacanthinae) from China. *Mitochondrial DNA Part B-Resources* 4**,** 284-285.

Wang, J.J., Yang, M.-F., Dai, R.-H., Li, H., and Wang, X.-Y. (2018). Characterization and phylogenetic implications of the complete mitochondrial genome of Idiocerinae (Hemiptera: Cicadellidae). *International Journal of Biological Macromolecules* 120**,** 2366-2372.

Wang, J.J., Dai, R.H., Li, H., and Zhan, H.P. (2017a). Characterization of the complete mitochondrial genome of Japanagallia spinosa and Durgades nigropicta (Hemiptera: Cicadellidae: Megophthalminae). *Biochemical Systematics and Ecology* 74**,** 33-41.

Wang, J.J., Li, H., and Dai, R.H. (2017b). Complete mitochondrial genome of Taharana fasciana (Insecta, Hemiptera: Cicadellidae) and comparison with other Cicadellidae insects. *Genetica* 145**,** 593-602.

Wu, Y.F., Dai, R.H., Zhan, H.P., and Qu, L. (2016). Complete mitochondrial genome of Drabescoides nuchalis (Hemiptera: Cicadellidae). *Mitochondrial DNA Part A* 27**,** 3626-3627.

Yu, P.F., Wang, M.X., Cui, L., Chen, X.X., and Han, B.Y. (2017). The complete mitochondrial genome of Tambocerus sp. (Hemiptera: Cicadellidae). *Mitochondrial DNA Part A* 28**,** 133-134.

Zhao, X., and Liang, A.-P. (2016). Complete DNA sequence of the mitochondrial genome of the treehopper Leptobelus gazella (Membracoidea: Hemiptera). *Mitochondrial DNA Part A* 27**,** 3318-3319.

Zhou, N., Wang, M., Cui, L., Chen, X.X., and Han, B.Y. (2016). Complete mitochondrial genome of Empoasca vitis (Hemiptera: Cicadellidae). *Mitochondrial DNA Part A* 27**,** 1052-1053.

**Table S3 Partition strategies and evolutionary models of PCG12RNA datasets used in analysis.**

| **Subset** | **Best Model** | **Length (bp)** | **Partition names** |
| --- | --- | --- | --- |
| 1 | GTR+I+G | 1,182 | nad2_pos1, atp6_pos1, nad6_pos1, atp8_pos1, cox3_pos1, cox2_pos1 |
| 2 | TVM+I+G | 1,982 | cox1_pos2, nad4l_pos2, nad1_pos2, cob_pos2, cox2_pos2, cox3_pos2, atp6_pos2 |
| 3 | TVM+I+G | 599 | nad3_pos2, nad6_pos2, atp8_pos2, nad2_pos2 |
| 4 | GTR+I+G | 885 | cob_pos1, cox1_pos1 |
| 5 | GTR+I+G | 1,454 | nad4_pos1, nad3_pos1, nad1_pos1, nad5_pos1, nad4l_pos1 |
| 6 | GTR+I+G | 940 | nad5_pos2, nad4_pos2 |
| 7 | GTR+I+G | 1,512 | 12S, 16S |

**Table S4 Partition strategies and evolutionary models of AA datasets used in analysis.**

| **Subset** | **Best Model** | **Length (bp)** | **Partition names** |
| --- | --- | --- | --- |
| 1 | MTART+I+G | 1,405 | atp6, cox1, cob, nad1 |
| 2 | MTMAM+I+G | 479 | cox2, cox3 |
| 3 | MTREV+I+G | 32 | atp8 |
| 4 | MTART+I+G+F | 1,441 | nad2, nad3, nad4, nad4l, nad5, |
| 5 | MTREV+I+G+F | 136 | nad6 |

**Table S5 Partition strategies and evolutionary models of PCGs datasets used in analysis.**

| **Subset** | **Best Model** | **Length (bp)** | **Partition names** |
| --- | --- | --- | --- |
| 1 | GTR+I+G | 768 | nad3_pos1, nad2_pos1, atp6_pos1, nad6_pos1 |
| 2 | GTR+I+G | 3,331 | nad5_pos2, nad4_pos2, cox2_pos2, cox1_pos2, nad2_pos2, atp6_pos2, nad3_pos2, nad4l_pos2, nad1_pos2, cox3_pos2, cob_pos2 |
| 3 | GTR+I+G | 2,133 | nad3_pos3, nad6_pos3, cox3_pos3, cox2_pos3, atp6_pos3, cox1_pos3, nad2_pos3, cob_pos3 |
| 4 | GTR+I+G | 1,574 | nad5_pos1, nad4l_pos1, nad6_pos2, atp8_pos2, atp8_pos1, atp8_pos3, nad4_pos1, nad1_pos1 |
| 5 | GTR+I+G | 1,365 | cox2_pos1, cox3_pos1, cob_pos1, cox1_pos1 |
| 6 | TIM+I+G | 1,335 | nad4l_pos3, nad4_pos3, nad1_pos3, nad5_pos3 |

**Table S6 Annotations of the mitogenomes of *Macropsis notata.***

| Gene | Strand | Position | Size (bp) | Anticodon | Start codon | Stop codon | Intergenic nucleotides* |
| --- | --- | --- | --- | --- | --- | --- | --- |
| *trnI* | J | 1-62 | 62 | 29-31 GAT |  |  |  |
| *trnQ* | N | 60-126 | 67 | 95-97 TTG |  |  | -3 |
| *trnM* | J | 126-193 | 68 | 157-159 CAT |  |  | -1 |
| *ND2* | J | 194-1159 | 966 |  | ATA | TAA | 0 |
| *trnW* | J | 1158-1222 | 65 | 1188-1190 TCA |  |  | -2 |
| *trnC* | N | 1216-1279 | 64 | 1247-1249 GCA |  |  | 3 |
| *trnY* | N | 1289-1351 | 63 | 1327-1329 GTA |  |  | 9 |
| *COX1* | J | 1350-2883 | 1534 |  | ATG | T | -2 |
| *trnL1* | J | 2884-2948 | 65 | 2913-2915 TAA |  |  | 0 |
| *COX2* | J | 2949-3628 | 680 |  | ATT | TA | 0 |
| *trnK* | J | 3629-3694 | 66 | 3660-3662 CTT |  |  | 0 |
| *trnD* | J | 3695-3759 | 65 | 3725-3727 GTC |  |  | 0 |
| *ATP8* | J | 3760-3912 | 153 |  | TTG | TAA | 0 |
| *ATP6* | J | 3906-4562 | 657 |  | ATG | TAA | -7 |
| *COX3* | J | 4563-5342 | 780 |  | ATG | TAA | 0 |
| *trnG* | J | 5343-5405 | 63 | 5373-5375 TCC |  |  | 0 |
| *ND3* | J | 5403-5759 | 357 |  | ATA | TAA | -3 |
| *trnA* | J | 5761-5826 | 66 | 5790-5792 TGC |  |  | 1 |
| *trnR* | J | 5826-5888 | 63 | 5855-5857 TCG |  |  | -1 |
| *trnN* | J | 5888-5953 | 66 | 5920-5922 GTT |  |  | -1 |
| *trnS1* | J | 5953-6010 | 58 | 5974-5976 GCT |  |  | -1 |
| *trnE* | J | 6010-6075 | 66 | 6041-6043 TTC |  |  | -1 |
| *trnF* | N | 6076-6139 | 64 | 6106-6108 GAA |  |  | 0 |
| *ND5* | N | 6140-7814 | 1675 |  | ATT | T | 0 |
| *trnH* | N | 7812-7872 | 61 | 7840-7842 GTG |  |  | -2 |
| *ND4* | N | 7873-9189 | 1317 |  | ATG | TAA | 0 |
| *ND4L* | N | 9183-9455 | 273 |  | ATG | TAG | -7 |
| *trnT* | J | 9458-9521 | 64 | 9490-9492 TGT |  |  | 2 |
| *trnP* | N | 9522-9584 | 63 | 9552-9554 TGG |  |  | 0 |
| *ND6* | J | 9587-10078 | 492 |  | ATT | T | 2 |
| *CytB* | J | 10071-11207 | 1137 |  | ATG | TAA | 2 |
| *trnS2* | J | 11207-11270 | 64 | 11237-11239 TGA |  |  | -1 |
| *ND1* | N | 11272-12202 | 931 |  | ATT | T | 1 |
| *trnL2* | N | 12203-12267 | 65 | 12236-12238 TAG |  |  | 0 |
| *12S* | N | 12268-13458 | 1191 |  |  |  | 0 |
| *trnV* | N | 13459-13522 | 64 | 13489-13491 TAC |  |  | 0 |
| *16S* | N | 13523-14255 | 733 |  |  |  | 0 |
| Control region |  | 14256-16323 | 2068 |  |  |  | 0 |

**Table S7 Annotations of the mitogenomes of *Oncopsis nigrofasciata.***

| Name | Direction | Location | Size(bp) | Start | Stop | Anticodon | Intergenic nucleotides |
| --- | --- | --- | --- | --- | --- | --- | --- |
| *trnI* | F | 1-63 | 63 | - | - | 30-32 GAT | 0 |
| *trnQ* | R | 127-61 | 67 | - | - | 97-95 TTG | -3 |
| *trnM* | F | 127-194 | 68 | - | - | 158-160 CAT | -1 |
| *ND2* | F | 195-1,160 | 966 | ATG | TAA | - | 0 |
| *trnW* | F | 1,159-1,226 | 68 | - | - | 1,190-1,192 TCA | -2 |
| *trnC* | R | 1,285-1,219 | 67 | - | - | 1,255-1,253 GCA | -8 |
| *trnY* | R | 1,358-1,296 | 63 | - | - | 1,329-1,327 GTA | -10 |
| *COX1* | F | 1,357-2,895 | 1,539 | ATG | TAA | - | -2 |
| *trnL1* | F | 2,891-2,955 | 65 | - | - | 2,920-2,922 TAA | -5 |
| *COX2* | F | 2,956-3,645 | 690 | ATA | TAG | - | 0 |
| *trnK* | F | 3,636-3,700 | 65 | - | - | 3,667-3,669 CTT | -10 |
| *trnD* | F | 3,701-3,765 | 65 | - | - | 3,731-3,733 GTC | 0 |
| *ATP8* | F | 3,766-3,918 | 153 | TTG | TAA | - | 0 |
| *ATP6* | F | 3,918-4,568 | 651 | ATA | TAA | - | -1 |
| *COX3* | F | 4,569-5,348 | 780 | ATG | TAG | - | 0 |
| *trnG* | F | 5,349-5,411 | 63 | - | - | 5,379-5,381 TCC | 0 |
| *ND3* | F | 5,412-5,765 | 354 | ATG | TAG | - | 0 |
| *trnA* | F | 5,764-5,831 | 68 | - | - | 5,793-5,795 TGC | -2 |
| *trnR* | F | 5,832-5,896 | 65 | - | - | 5,861-5,863 TCG | 0 |
| *trnN* | F | 5,898-5,963 | 66 | - | - | 5,928-5,930 GTT | 1 |
| *trnS1* | F | 5,963-6,020 | 58 | - | - | 5,985-5,987 GCT | -1 |
| *trnE* | F | 6,024-6,087 | 64 | - | - | 6,054-6,056 TTC | 3 |
| *trnF* | R | 6,156-6,094 | 63 | - | - | 6,124-6,122 GAA | 6 |
| *ND5* | R | 7,833-6,160 | 1,674 | TTG | TAA | - | 3 |
| *trnH* | R | 7,904-7,834 | 71 | - | - | 7,866-7,864 GTG | 0 |
| *ND4* | R | 9,223-7,904 | 1,320 | ATA | TAA |  | -1 |
| *ND4L* | R | 9,501-9,220 | 282 | ATT | TAA | - | -4 |
| *trnT* | F | 9,494-9,560 | 67 | - | - | 9,528-9,530 TGT | -8 |
| *trnP* | R | 9,624-9,561 | 64 | - | - | 9,593-9,591 TGG | 0 |
| *ND6* | F | 9,627-10,118 | 492 | ATC | TAA | - | 2 |
| *Cytb* | F | 10,123-11,247 | 1,125 | ATA | TAG | - | 4 |
| *trnS2* | F | 11,250-11,312 | 63 | - | - | 11,281-11,283 TGA | 2 |
| *ND1* | R | 12,247-11,303 | 945 | ATA | TAA | - | -10 |
| *trnL2* | R | 12,309-12,245 | 65 | - | - | 12,280-12,278 TAG | -3 |
| *16S* | R | 13,501-12,310 | 1,192 | - | - | - | 0 |
| *trnV* | R | 13,563-13,502 | 62 | - |  | 13,533-13,531 TAC | 0 |
| *12S* | R | 14,311-13,564 | 748 | - | - | - | 0 |
| Control region | - | 14,312-15,927 | 1,616 | - | - | - | 0 |
